# Intergenerational plasticity to cycling high temperature and hypoxia affects offspring stress responsiveness and tolerance in zebrafish

**DOI:** 10.1242/jeb.245583

**Published:** 2023-08-21

**Authors:** Michael Y.-T. Lim, Nicholas J. Bernier

**Affiliations:** Department of Integrative Biology, University of Guelph, 50 Stone Road East, Guelph, ON N1G 2W1, Canada

**Keywords:** Non-genetic inheritance, Environmental stressors, Cortisol, Heat shock proteins, Thermal tolerance, Hypoxia resistance

## Abstract

Predicted climate change-induced increases in heat waves and hypoxic events will have profound effects on fishes, yet the capacity of parents to alter offspring phenotype via non-genetic inheritance and buffer against these combined stressors is not clear. This study tested how prolonged adult zebrafish exposure to combined diel cycles of thermal stress and hypoxia affect offspring early survival and development, parental investment of cortisol and heat shock proteins (HSPs), larval offspring stress responses, and both parental and offspring heat and hypoxia tolerance. Parental exposure to the combined stressor did not affect fecundity, but increased mortality, produced smaller embryos and delayed hatching. The combined treatment also reduced maternal deposition of cortisol and increased embryo *hsf1*, *hsp70a*, HSP70, *hsp90aa* and HSP90 levels. In larvae, basal cortisol levels did not differ between treatments, but acute exposure to combined heat stress and hypoxia increased cortisol levels in control larvae with no effect on larvae from exposed parents. In contrast, whereas larval basal *hsf1*, *hsp70a* and *hsp90aa* levels differed between parental treatments, the combined acute stressor elicited similar transcriptional responses across treatments. Moreover, the combined acute stressor only induced a marked increase in HSP47 levels in the larvae derived from exposed parents. Finally, combined hypoxia and elevated temperatures increased both thermal and hypoxia tolerance in adults and conferred an increase in offspring thermal but not hypoxia tolerance. These results demonstrate that intergenerational acclimation to combined thermal stress and hypoxia elicit complex carryover effects on stress responsiveness and offspring tolerance with potential consequences for resilience.

## Teaser

**Summary:** Parental exposure to cycles of elevated temperatures and hypoxia confers complex carryover effects that may promote resilience to environmental stressors in larval zebrafish.

## INTRODUCTION

In aquatic environments, the combined climate-change-induced stressors of high temperature and low oxygen levels (hypoxia) are expected to have profound effects on fishes. In part, the challenges posed by this combined environmental stressor stems from the various interacting effects of elevated water temperatures and hypoxia (Earhart et al., 2022). Most importantly, high temperatures increase the metabolic demand of microorganisms such as bacteria and algae, which can drastically decrease water oxygen levels (Iriberri et al., 1985; Pörtner and Knust, 2007; Schulte, 2015; Earhart et al., 2022). The increased frequency of heat waves and extreme hypoxic events linked with climate change and anthropogenically driven eutrophication is expected to reduce fish performance (e.g. development, growth, reproduction and survival) and their overall biomass (Díaz and Rosenberg, 2011; Jenny et al., 2016; Sampaio et al., 2021; Harvey et al., 2022). Beyond extreme events, climate-change-driven increases in temperature are increasing the prevalence, severity and variability of environmental hypoxia, conditions that are negatively impacting offspring development and recruitment (Smith and Able, 2003; Cheek et al., 2009; Stierhoff et al., 2009; Baumann et al., 2015; Campbell and Rice, 2014; Tommasi et al., 2015; Rooper et al., 2019; Mikloska et al., 2022). Therefore, there is an urgent need to understand the capacity of fishes to adjust their phenotype and buffer against the combined stressor of high temperature and hypoxia.

Resilience to climate change in fishes may be affected by intergenerational plasticity, i.e. through the capacity of parents to affect the tolerance of their progeny. For example, parental heat exposure can increase offspring aerobic scope in spiny chromis (*Acanthochromis polyacanthus*; Donelson et al., 2012) and heat tolerance in rainbow trout (*Oncorhynchus mykiss*; Butzge et al., 2021). Depending on exposure duration, parental hypoxia exposure can either increase or decrease offspring hypoxia tolerance in zebrafish (*Danio rerio*; Ho and Burggren, 2012). If offspring are exposed to conditions mismatched to parental environments, intergenerational effects can also be maladaptive (Schade et al., 2014; Bautista and Burggren, 2019). In general, offspring phenotypes can be modified by altering maternal (Mousseau and Fox, 1998) and/or paternal (Jiang et al., 2013; Shama and Wegner, 2014; Valdivieso et al., 2020) investments (reviewed by Sheriff and Love, 2013; Donelson et al., 2018; Earhart et al., 2022) and by epigenetic mechanisms (Beldade et al., 2011; Munday, 2014; Donelson et al., 2018; Ryu et al., 2018; Luu et al., 2021). To date, however, very few studies (if any) have determined how parental exposure to combined high temperature and hypoxia affects progeny heat and hypoxia tolerance or stress responsiveness. Similarly, the mechanisms involved in mediating intergenerational plasticity in response to the combined stressor of high temperature and hypoxia are largely unknown.

Among the substances involved in non-genetic inheritance between parents and offspring, both glucocorticoids (GCs) and heat shock proteins (HSPs) are potential candidates for shaping offspring phenotype in response to environmental stressors. In placental and egg-laying species, maternal stressors can increase the deposition of GCs into offspring (Saino et al., 2005; Almasi et al., 2012; Sheriff and Love, 2013; Sopinka et al., 2017a), and maternal GCs are known for their capacity to induce epigenetic modifications and shape offspring morphology, physiology and behavior (McCormick, 1998; Kapoor and Matthews, 2005; Eriksen et al., 2011; Capelle et al., 2016; Sopinka et al., 2017b; Best et al., 2017). In fish, however, it is unclear whether maternal stressors lead to an increase in offspring GC levels (Stratholt et al., 1997; Sopinka et al., 2014; Taylor et al., 2016; Lim and Bernier, 2022; Magierecka et al., 2022) or whether exogenous GCs can improve offspring stressor tolerance (Redfern et al., 2017; Warriner et al., 2020a,b). In addition, fish eggs may buffer against maternally derived GCs using ATP-binding cassette efflux transporters (Fischer et al., 2013; Paitz et al., 2016) and by converting GCs into inert forms using the catabolic enzymes 11β hydroxysteroid dehydrogenase type 2 (11β-HSD2) and 20β-HSD2 (Alderman and Vijayan, 2012; Tokarz et al., 2012; Faught et al., 2016). In teleosts, exposure to heat stress and/or hypoxic conditions can lead to an increase in the production of specific inducible HSPs, including HSP90α (encoded as *hsp90aa*), HSP70 (encoded as *hsp70a*) and HSP47 (Krone and Sass, 1994; Delaney and Klesius, 2004; Todgham et al., 2005; Vinagre et al., 2012; Narum et al., 2013; Chadwick and McCormick, 2017; Levesque et al., 2019; Lim and Bernier, 2022). Interestingly, maternal transfer of HSPs is associated with increases in progeny thermal tolerance in both *Artemia* (Norouzitallab et al., 2014) and *Drosophila* (Lockwood et al., 2017). In zebrafish, parental exposure to chronic diel cycles of heat stress and/or hypoxia can affect the embryonic deposition of GCs and inducible HSPs (Lim and Bernier, 2022). Whether this dynamic parental progeny investment of GCs and HSPs is associated with intergenerational plasticity remains to be determined.

Therefore, the objectives of this study were to determine whether adult zebrafish exposure to diel cycles of heat stress and hypoxia affect: (1) offspring early survival and development, (2) parental investment of GCs and HSPs, (3) larval offspring endocrine and cellular stress responses to combined heat stress and hypoxia, and (4) parental and larval offspring heat and hypoxia tolerance. Zebrafish were selected for this study as they experience variable temperatures (∼6–38°C) and O_2_ levels (∼16–350% dissolved O_2_) in their native habitat (Khan et al., 1970; Engeszer et al., 2007; Spence et al., 2008). Consistent with the environmental/maternal-matching hypothesis, which states that maternal stress can be adaptive if the parental and offspring environment match (Sheriff and Love, 2013), we predicted that in response to altered parental investments, zebrafish larvae derived from parents exposed to diel cycles of heat stress and hypoxia would have blunted endocrine and cellular stress responses to a combined elevated temperature and hypoxia stressor, as well as an improved tolerance to heat and hypoxia.

## MATERIALS AND METHODS

### Experimental animals

Adult zebrafish, *Danio rerio* (F. Hamilton 1822), were acquired from AQuality Tropical Fish (Mississauga, ON, Canada). The *F*_0_ generation was reared in a recirculating multi-tank system (ZebTEC rack, Tecniplast USA, West Chester, PA, USA) for at least 3 months after acquisition, and maintained on a 12 h:12 h light:dark cycle, at ∼27.5°C under normoxic conditions (>85% dissolved oxygen, DO) and pH ∼7.2. Adult zebrafish were held in 3.5 l tanks at a density of ∼25 fish per tank and fed twice daily to satiation with 0.5 mm sinking pellets (Northfin, Toronto, ON, Canada) and once with brine shrimp (Hikari USA, Hayward, CA, USA). All experiments were performed in accordance with guidelines set by the Canadian Council for Animal Care and were approved by the University of Guelph's Animal Care Committee.

### Experimental design

A total of 108 adult *F*_0_ fish (54 per treatment group) were separated based on secondary sexual characteristics into 12 female baskets (6 per basket) and 4 male baskets (9 per basket). Baskets were separated and submerged into two ∼180 l aquaria, each with ∼120 l of water. Each aquarium contained 6 female and 2 male baskets arranged randomly in two rows and four columns. Tanks were supplied with deionized City of Guelph tap water (adjusted to pH ∼7.1 with Discus and Malawi/Victoria Buffers; Seachem Laboratories, Madison, GA, USA) combined with a sea salt mixture (0.5 g l^−1^; Instant Ocean, Spectrum Brands, Blacksburg, VA, USA), and maintained on a 12 h:12 h light:dark cycle at 27°C and aerated (>85% DO; >6.77 mg O_2_ l^−1^). All tanks were equipped with a ∼113 l sponge filter. Water temperature and % DO were measured every second with an automated monitoring and control system (Argus Control Systems Ltd, Surrey, BC, Canada). Throughout the experiment, temperature and DO readings were verified with a hand-held meter (Handy Polaris; Oxyguard, Farum, Denmark). Water changes (∼20%) were performed daily, and pH (Accumet Basic 15; Thermo Fisher Scientific, Waltham, MA, USA) and nitrogenous waste tests (API Freshwater Water Master Test Kit; API, Chalfont, PA, USA) were performed twice weekly to identify and correct for any deviation from initial water quality conditions. Each treatment group was carried out a total of five times for a total of 180 females and 90 males per treatment.

Fish were initially held under control conditions (27°C, ∼85% DO) to habituate for 3 days, and then exposed to one of two treatments for 14 days: (1) control conditions (mean±s.d., 26.98±0.03°C and 85.60±1.27% DO; Fig. S1A) and (2) cycling temperature and hypoxia (combined exposure; Fig. S1B). To control for possible variation in parental exposure conditions (e.g. light intensity, proximity to airstones), spawning baskets in the ∼180 l aquaria were rotated without emersion one position clockwise daily, and fish were fed thrice daily with floating flake food (TetraMin Tropical Flakes, Blacksburg, VA, USA) throughout the habituation and treatment periods. In the combined exposure treatment, warming began with light phase onset (27°C, 08:00 h), until reaching and holding the maximum temperature (36°C) until cooling began with the onset of the dark phase (36°C, 20:00 h). Warming/cooling rates were ∼1°C h^−1^ across 9 h, with an average temperature of 31.49±3.16°C. Scaled to water temperatures, % DO was gradually lowered using a mixture of compressed air and N_2_ gas until it reached 30% (2.38 mg l^−1^ at 27°C) during the dark phase, and raised to 85% (5.81 mg l^−1^ at 36°C) during the light phase. Changes in % DO were ∼7.2% h^−1^, with an average of 56.44±18.47% DO.

#### Effects of parental exposure to cycling elevated temperatures and hypoxia

##### Parental fecundity, offspring size, cumulative mortality and cumulative hatch

To breed the *F*_0_ generation, on the evening of the 13th day of the treatment period, three males from each treatment group were added to each female basket for a total of six mixed-sex baskets per treatment. On the morning of the 14th day, eggs were collected, cleaned and counted, and dead embryos were removed. The average fecundity and survival (percentage of live embryos/total number of embryos) were determined per female per basket for each treatment group to quantify measures of spawning success. Using images taken with a zoom stereo microscope (Z30 V, Leica, Buffalo, NY, USA) and ImageJ (version 1.52a, National Institutes of Health, Bethesda, MD, USA), average egg and yolk size of live 1 h post-fertilization (hpf) embryos were determined to quantify measures of parental reproductive investment. Through the positioning of a 1 mm scale in each image, pixel measurements obtained using the straight measurement tool in ImageJ were converted into mm measurements. Owing to the two-dimensional nature of the images taken, egg and yolk size are represented by their maximum cross-sectional area calculated using two perpendicular diameters and using the equation for an ellipsoid shape [area=π(*L*/2×*W*/2), where *L* is the length and *W* is the width of each region; Fig. S2]. A total of 20 random embryos from each treatment were chosen for imaging. To ensure even sampling, for each treatment, five eggs were randomly selected from four spawning baskets. A yolk/egg ratio was calculated for each embryo to determine the relative amount of yolk deposited in each embryo. A separate subset of live embryos was snap frozen on dry ice prior to storage at −80°C for subsequent analyses. Remaining embryos were randomly mixed within treatments and transferred into Petri dishes (15×100 mm) with egg water (60 µg ml^−1^ Instant Ocean; 0.0004% Methylene Blue mixed with deionized water, pH 7), at a density of ∼75 embryos per dish, and kept at 28°C. At 8 hpf, dishes were checked for dead or hatched embryos, with dead embryos removed, and live embryos imaged to quantify egg and yolk size as above. Similar mortality and hatch checks were performed within ∼45 min of first light between 1 and 5 days post-fertilization (dpf), after which each dish was given a ∼80% water change. At 5 dpf, a subset of larvae was anesthetized with tricaine mesylate (MS-222, ∼0.02%; Syndel International, Qualicum Beach, BC, Canada) and imaged to determine body length (tip of tail to mouth; Table 1).

**Table 1.**
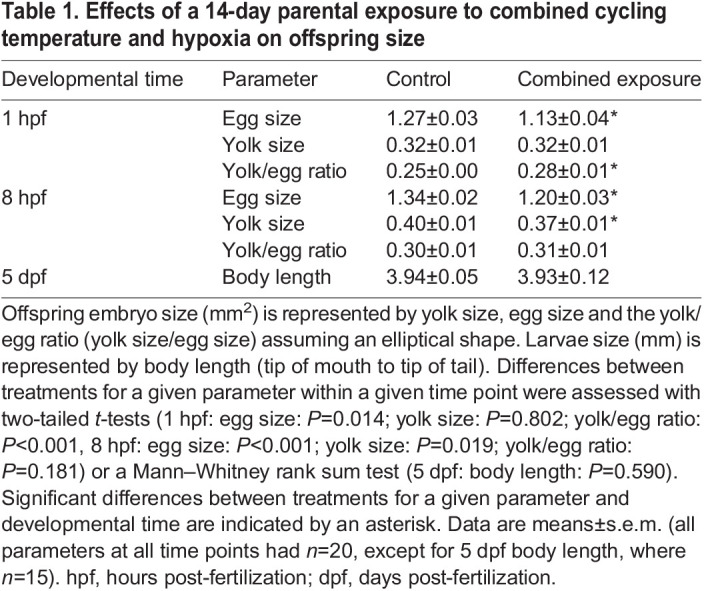
Effects of a 14-day parental exposure to combined cycling temperature and hypoxia on offspring size

##### Offspring endocrine and cellular stress responses

To assess whether the 14 day combined exposure treatment elicited a transfer of parental signals into their offspring, and whether these contributions to non-genetic inheritance have lasting effects on the early development of the endocrine and cellular stress responses, we quantified embryonic (1 hpf) and larval (5 dpf) cortisol levels, mRNA levels (embryo: *hsd11b2*, *hsd20b2*, *abcb4*, *hsf1*, *hsp70a*, *hsp90aa* and *hsp47*; larvae: same as embryo but omitting *abcb4*) and protein levels (embryo and larvae: HSP70, HSP90 and HSP47). At 1 hpf, cortisol, mRNA and protein levels were quantified from pools of 30 embryos (sampled before zygotic transcription; Kane and Kimmel, 1993). At 5 dpf, cortisol, mRNA and protein levels were quantified from pools of 15 larvae.

To assess whether parental exposure to diel cycling heat and hypoxia alters the response of their larvae to these environmental stressors, we exposed 5 dpf larvae to a combined elevated temperature and hypoxia stressor. Specifically, for each parental exposure, two 6-well plates of larvae (15 larvae per well) were prepared on the morning of 4 dpf and supplied with egg water (60 µg ml^−1^ Instant Ocean, pH 7). On the morning of 5 dpf, each well was given an 80% water change and the larvae from one 6-well plate were transferred into a custom-made 6-well plate. Customization of the 6-well plate included removal of well bottoms and addition of ∼300 µm mesh bottoms. The custom plate was imbedded in a plexiglass lid enclosing a ∼30 l container filled with egg water (as above). The water within the container was heated to 36°C (verified with a Traceable Hi-Accuracy Dual Thermometer; VWR International) via a circulation bath and an external coil. The DO level was maintained at ∼30% DO using bubbled N_2_ (∼2.04 mg l^−1^; OxyCTRL software, Loligo Systems, Tjele, Denmark). Three magnetic stir bars were strategically placed within the container to ensure good water mixing. As such, the combined exposure was immediate and maintained for 20 min. The other 6-well plate of larvae per parental exposure served as a control ‘unstressed group’, where larvae were transferred into a new standard 6-well plate and returned to a 28°C incubator. After 20 min, both sets of larvae were anesthetized with MS-222 (∼0.02%), snap-frozen on dry ice and then stored at −80°C prior to whole-body cortisol analysis. The combined exposure (36°C, ∼30% DO) experiment outlined above was performed an additional three times to assess control and stress-induced mRNA and protein levels in larvae from each parental treatment.

To assess whether parental exposure to diel cycling heat and hypoxia alters the response of their larvae to a novel stressor, we exposed 5 dpf larvae to a previously validated swirling stressor (Williams et al., 2017). Briefly, on the morning of 4 dpf, larvae from each parental exposure were placed in two 6-well plates (15 larvae per well) with 5 ml of egg water (60 µg ml^−1^ Instant Ocean, pH 7) and returned to a 28°C incubator. On the morning of 5 dpf, each well was given an 80% water change, with one 6-well plate placed in a heated orbital platform shaker (28°C, 0 rpm) and the other 6-well plate returned to a 28°C incubator to serve as control. After a 2 h habituation period, the larvae were exposed to 0 or 180 rpm for 20 min. After the exposure, both sets of larvae were anesthetized with MS-222 (∼0.02%), snap-frozen on dry ice and then stored at −80°C prior to whole-body cortisol analysis.

##### Parental and offspring heat and hypoxia tolerance

To assess whether the 14-day parental exposure alters parental heat and hypoxia tolerance, a total of 48 adult zebrafish were exposed to either control or the combined exposure treatment as described above. Adult fish were separated into baskets at a density of six females or six males (four baskets per gender). Each treatment aquarium contained two female and two male baskets arranged randomly into two rows and two columns for each treatment aquarium. Each treatment group was repeated an additional time for a total of 48 females and 48 males per treatment. Fish were initially held under control conditions (27°C, ∼85% DO) to habituate for 3 days, and then exposed to one of the two treatments. Fish were fed and spawning baskets were rotated as described above throughout the habituation and treatment periods, except for the final 24 h before tolerance tests (morning of the 13th day to testing day). To assess adult zebrafish heat tolerance, a critical thermal maximum (CT_max_) test was performed. On the morning of the 14th treatment day, shortly after first light (∼08:15 h), two female and two male baskets (24 fish total) were moved to a new aquarium with ∼30 l of deionized City of Guelph tap water (adjusted to pH ∼7.1) combined with a sea salt mixture (0.5 g l^−1^). This aquarium was held at 27°C (heaters controlled by Argus) and aerated to >85% DO. Temperature was verified with a handheld digital thermometer (Traceable Hi-Accuracy Dual Thermometer; VWR International) and % DO was verified with a hand-held meter (Handy Polaris; Oxyguard). At 11:00 h, water temperature was increased at a rate of 0.29°C min^−1^ until all fish had lost equilibrium. When a fish lost equilibrium, the current temperature was recorded, and the fish was placed in a separate 27°C aquarium to recover. To assess adult zebrafish hypoxia tolerance, a time to loss of equilibrium (LOE) test was performed. On the morning of the 14th treatment day, shortly after first light (∼08:15 h), two female and two male baskets (24 fish total) were moved to a new aquarium with ∼120 l deionized City of Guelph tap water (adjusted to pH ∼7.1) combined with a sea salt mixture (0.5 g l^−1^). This aquarium was held at 27°C and aerated to >85% DO (heaters and aeration controlled by Argus). Baskets were kept ∼1 cm below the water's surface to prevent surface respiration, and transparent plexiglass sheets were placed at the water's surface to reduce mixing with ambient air. Temperature and DO readings were verified with a hand-held meter (Handy Polaris; Oxyguard). At 11:00 h, % DO was decreased at a rate of ∼0.9% min^−1^ until it reached 8% DO, and then maintained at 8% until all fish had lost equilibrium. Owing to one fish losing equilibrium at 14.7% DO (1.17 mg l^−1^), time to LOE in adult fish was recorded as the time elapsed between 16.4% DO (1.30 mg l^−1^) and the fish losing equilibrium. When a fish lost equilibrium, the time was recorded and the fish was placed in a separate 27°C, >85% DO aquarium to recover.

To assess whether the 14-day parental exposure affects larval progeny heat tolerance, a CT_max_ test was performed at 5 dpf for each parental treatment as per Andreassen et al. (2022). Briefly, on the morning of 4 dpf, 24 larvae were placed individually into two 12-well plates. Each well was supplied with 750 μl of egg water (60 µg ml^−1^ Instant Ocean, pH 7). Each 12-well plate was kept at 28°C in a heated incubator until 5 dpf. On the morning of 5 dpf, shortly after first light (∼08:15 h), the larvae from one 12-well plate were transferred into a custom-made 12-well plate suspended in an aluminum foil tin. Customization of the 12-well plate included removal of well bottoms and addition of cell culture inserts with ∼300 µm mesh bottoms. The tin was filled with egg water (as above) and supplied with a magnetic stir bar to ensure water mixing. The tin was placed on a heated stir plate (Fisherbrand Isotemp Hot Plate Stirrer; Thermo Fisher Scientific) and kept at 28°C (verified with a Traceable Hi-Accuracy Dual Thermometer; VWR International). At 09:30 h, water temperature was increased at a rate of ∼0.28°C min^−1^ until all larvae lost equilibrium. As the temperature increased, larvae became more sluggish but still responded to a tapping stimulus from a pipette. This temperature was recorded as their ‘reduced movement temperature’. The temperature at which a larva did not respond to a tapping stimulus from a pipette for 3 s was recorded as their CT_max_ (temperature at initial non-responsiveness). When a larva lost equilibrium, they were placed in a separate Petri dish with fresh egg water (60 µg ml^−1^ Instant Ocean, pH 7) to recover. Once recovery from the CT_max_ test was confirmed, all larvae were euthanized with an overdose of MS-222 (∼0.02%). After replacing the egg water and providing a new group of 5 dpf larvae a 75 min habituation period, a second CT_max_ test was performed at 13:30 h. Because the CT_max_ values for a given treatment did not differ between testing times (data not shown), CT_max_ data were pooled within treatments.

To assess whether the 14-day parental exposure affects larval progeny hypoxia tolerance, a time to LOE test was performed at 5 dpf for each treatment as per Ho and Burggren (2012). Briefly, on the morning of 4 dpf, 16 larvae were separated individually into two 12-well plates (eight larvae per 12-well plate). Each occupied well was supplied with 750 μl of egg water (60 µg ml^−1^ Instant Ocean, pH 7). Each 12-well plate was kept at 28°C in a heated incubator until 5 dpf. On the morning of 5 dpf (∼11:00 h), each larva from one 12-well plate were transferred into an individual custom-made 20 ml glass vial. Each vial was filled with egg water (as above) supplied by a 16-channel peristaltic cassette pump (Watson-Marlow Fluid Technology Group, Falmouth, UK) at 1.0 ml min^−1^. Egg water supplied to each glass vial was warmed to 28°C (verified with a Traceable Hi-Accuracy Dual Thermometer; VWR International) and bubbled with N_2_ to ∼20% DO (∼1.56 mg l^−1^; controlled through OxyCTRL software, Loligo Systems). As such, the hypoxia exposure was immediate and maintained until all larvae lost equilibrium. The time at which a larva did not respond to a tapping stimulus on the outside of the glass vial for 3 s was recorded as their time to LOE. Once the time to LOE was recorded, the larvae were placed in a separate Petri dish with fresh egg water (as above) to recover. Once recovery from the time to LOE test was confirmed, all larvae were euthanized with an overdose of MS-222 (∼0.02%). After replacing the egg water and providing a new group of 5 dpf larvae a 75 min habituation period, a second time to LOE test was performed at 13:30 h. Because the LOE values for a given treatment did not differ between testing times (data not shown), time to LOE data were pooled within treatments.

### Analytical techniques

#### Steroid extraction and quantification

Embryos and larvae were processed as described in Lim and Bernier (2022). Briefly, samples were homogenized and extracted twice with MeOH prior to C_18_ column purification (100 mg octadecyl [C_18_], 1 ml column, Agela Technologies, Tianjin, China). Samples recovered from the C_18_ columns were dried and reconstituted in 110 µl of diluted extraction buffer (as per the manufacturer's instructions; Neogen, Lexington, KY, USA).

Embryo and larvae cortisol levels were quantified using a commercial ELISA kit (Neogen). All standards and samples were run in duplicate. Note that the slope generated from a serially diluted pool of embryos ran parallel to the standard curve. The intra- and inter-assay coefficients of variation were 9.3% (*n*=4) and 9.5% (*n*=3), respectively. The lower detection limit of the assay is 10 pg ml^−1^ as determined by Lim and Bernier (2022). According to the manufacturer, the cross-reactivity of the commercial antibody to other steroids is as follows: prednisolone 47.4%, cortisone 15.7%, 11-deoxycortisol 15.0%, prednisone 7.83%, corticosterone 4.81%, 6β-hydroxycortisol 1.37%, 17-hydroxyprogesterone 1.36% and deoxycorticosterone 0.94%. Steroids with cross-reactivity ≤0.06% are not presented.

#### RNA extraction, cDNA synthesis and mRNA quantification

Quantification of mRNA levels was completed via real-time PCR as per Lim and Bernier (2022). Briefly, embryo and larvae samples were homogenized in 0.5 ml of Ribozol RNA extraction reagent (Thermo Fisher Scientific) with a bead beater (Precellys Evolution, Bertin Technologies, Montigny-le-Bretonneux, France). To increase recovery, 10 µg of RNA-grade glycogen (Thermo Fisher Scientific) was added to each sample before precipitation in isopropanol at −80°C overnight. Total RNA was quantified via a nanodrop spectrophotometer (Nanodrop 2000 UV-vis; Thermo Fisher Scientific). From each sample, 100 ng was treated with DNase (Quanta Biosciences, Beverly, MA, USA) and used to synthesize cDNA using Quanta qScript (Quanta Biosciences) as per the manufacturer's instructions. Separate samples were treated identically without the addition of reverse transcriptase or without the presence of RNA to verify the absence of genomic DNA or contaminated reagents.

Quantitative real-time PCR was performed on a CFX96 system (Bio-Rad, Hercules, CA, USA) using 20 µl reactions that contained 10 µl of master mix (SsoAdvanced Universal SYBR Green Supermix, Bio-Rad), 5 µl of 10-fold diluted first-strand cDNA template or no-RT controls, and 2.5 µl of both forward and reverse primers (0.4 µmol l^−1^; Table S1). Default cycling conditions were used and followed by a melting curve analysis to verify the specificity of each PCR product. Samples were analyzed in triplicate and verified to have unimodal dissociation curves that matched the predicted melting point temperatures. To account for differences in amplification efficiency, standard curves were constructed for each gene using known dilutions of cDNA from ovary or embryo samples. Input values for each gene were obtained by fitting average cycle threshold (*C*_t_) values to the antilog of the gene-specific standard curves, thereby correcting for differences in amplification efficiency. To correct for any template input and/or transcriptional efficiency differences, input values were normalized to the geometric mean of the two housekeeping genes: elongation factor 1α (*ef1α*) and ribosomal protein L13A (*rpl13a*). Gene expression data are reported as fold-change relative to the control treatment mean value.

#### Protein quantification

Soluble protein was extracted from embryos and larvae as described in Lim and Bernier (2022). Briefly, samples (pools of 30 embryos or 15 larvae) were homogenized with radio immune-precipitation assay lysis buffer (300 µl for embryos, 150 µl for larvae) with protease inhibitors (0.574 mmol l^−1^ PMSF, 2 mmol l^−1^ EDTA) using a bead beater (Precellys Evolution, Bertin Technologies). Samples were mixed and centrifuged, and protein concentration in the supernatant was determined with a Bradford assay (Bio-Rad Protein Assay Dye Reagent, Bio-Rad). Samples were diluted to 1.98 µg µl^−1^, combined with 4× Laemmli buffer to a final concentration of 1.49 µg µl^−1^, vortexed, then incubated at 65°C for 10 min before being pulse spun and stored at −20°C.

Gel electrophoresis was performed on diluted samples as described in Lim and Bernier (2022). Briefly, samples were run alongside a protein ladder (PageRuler prestained protein ladder, Thermo Fisher Scientific), standards and a blank. The standards consisted of rat recombinant HSP70/HSP72 (cat. no. ADISPP7580, Enzo Life Sciences, Farmingdale, NY, USA) and native human HSP90 (cat. no. ADISPP770D, Enzo Life Sciences). A commercial HSP47 standard was not available, so a positive control made from a pool of gill tissues from heat-stressed adult zebrafish was used (validated previously by Lim and Bernier, 2022). The protein in each gel lane was separated on an 8% SDS-poly-acrylamide gel (and a 5% stacking gel) and transferred to a polyvinylidene difluoride (PVDF) membrane (Immobilon-P, Merck Millipore Ltd, Carrigtwohill, County Cork, Ireland). Membranes were blocked for 1 h at 20°C in 5% milk powder dissolved in Tris-buffered saline with Tween 20 (TBST). Incubations with primary antibody against HSP47 (1:1000; polyclonal rabbit HSP47/SERPINH1, cat. no. 20R-1310, Fitzgerald Industries International), HSP70 (1:5000; polyclonal rabbit HSP70/HSC70, cat. no. AS05083A, Agrisera, Vännäs, Sweden) and HSP90 (1:2500; mono clonal mouse HSP90, cat. no. SMC-107, StressMarq Biosciences Inc., Victoria, BC, Canada) were performed overnight at 4°C. According to the manufacturers, the HSP70 antibody recognizes both the inducible (HSP70) and constitutive (HSC70) isoforms, and the HSP90 antibody primarily recognizes the beta isoform (HSP90β) but may also detect the alpha isoform (HSP90α). Secondary antibody incubations for HSP47/HSP70 (1:20,000; polyclonal anti-rabbit goat, cat. no. AS09602, Agrisera) and HSP90 (1:5000 polyclonal anti-mouse goat, cat. no. ab5870, Abcam, Cambridge, UK) were performed for 1 h at 20°C. All antibodies were diluted in 1% milk powder dissolved in TBST.

Chemiluminescent detection of protein bands was performed using Superbright ECL (cat. no. AS16ECL-S, Agrisera). Blots were imaged using a Bio-Rad ChemiDoc MP Imaging System (Universal Hood III, Bio-Rad) and analyzed with ImageJ. Note that equal proteins were loaded on each gel and verified visually via Coomassie staining of PVDF membranes after immunodetection (Welinder and Ekblad, 2011). As specific concentrations were not of interest, all band densities are expressed relative to control tissues for each protein.

### Statistical analyses

All data are presented as means±s.e.m. unless otherwise stated. Differences between treatments were analyzed by two-tailed *t*-tests or two-way ANOVAs followed by a Holm–Šidák *post hoc* test when the ANOVA was significant. Square root or log_10_ transformation was applied if a Shapiro–Wilk test for normality or Levene's equal variance test among groups was significant. If transformation proved insufficient to meet both assumptions, a Mann–Whitney rank sum test was used instead of a two-tailed *t*-test, and Kruskal–Wallis one-way ANOVAs followed by Tukey *post hoc* tests were used instead of a two-way ANOVA across developmental time for cumulative mortality (8 hpf–5 dpf) and cumulative hatch (0–5 dpf) within parental treatments. Differences between parental treatments within offspring age were compared with subsequent two-tailed *t*-tests or Mann–Whitney rank sum tests (if assumptions were not met), with α=0.008 (Bonferroni's correction). For datasets comparing the effects of offspring exposure at 5 dpf within or between parental treatments, two-tailed *t*-tests or Mann–Whitney rank sum tests were used, with α=0.025 (Bonferroni's correction). Any outliers that were determined to be greater than or less than the 1.5×inter-quartile range from the upper quartile or lower quartile, respectively, were removed from gene and protein expression datasets (no more than two outliers were found in any one treatment group and was attributed to sample degradation). All tests were conducted in SigmaPlot 12.5 (SYSTAT Software, San Jose, CA, USA) and all other α were set at 0.05.

## RESULTS

### Effects of parental exposure to cycling elevated temperatures and hypoxia

#### Parental fecundity, offspring size, cumulative mortality and cumulative hatch

Relative to the control treatment, parental exposure to cycling temperature and hypoxia for 2 weeks had no effect on fecundity (embryos per female; control: 24.9±5.4, combined exposure: 32.6±7.3, Mann–Whitney rank sum test, *U*=428.50, *n*_1_=*n*_2_=30, *P*=0.754) or on the percentage of living embryos at 1 hpf (control: 91.8±3.0%, combined exposure: 97.5±0.7%, Mann–Whitney rank sum test, *U*=217.00, *n*_1_=21, *n*_2_=23, *P*=0.569). However, relative to the control treatment, embryos from combined exposure parents had 11% smaller eggs at 1 hpf (Table 1; two-tailed *t*-test, *t*_38_=2.59, *P*=0.014), no change in yolk size (two-tailed *t*-test, *t*_38_=0.25, *P*=0.802) and a higher yolk/egg ratio (1.1-fold, two-tailed *t*-test, *t*_38_=–3.61, *P*<0.001). At 8 hpf, embryos from combined exposure parents were smaller in size (10.4% smaller, two-tailed *t*-test, *t*_38_=4.43, *P*<0.001), with smaller yolk size (7.5% smaller, two-tailed *t*-test, *t*_38_=2.44, *P*=0.019), and no difference in yolk/egg ratio relative to embryos from control parents (two-tailed *t*-test, *t*_38_=1.36, *P*=0.181). At 5 dpf, larvae from both parental treatments did not differ in body length (Mann–Whitney rank sum test, *U*=99.00, *n*_1_=*n*_2_=15, *P*=0.590).

**Fig. 1.**
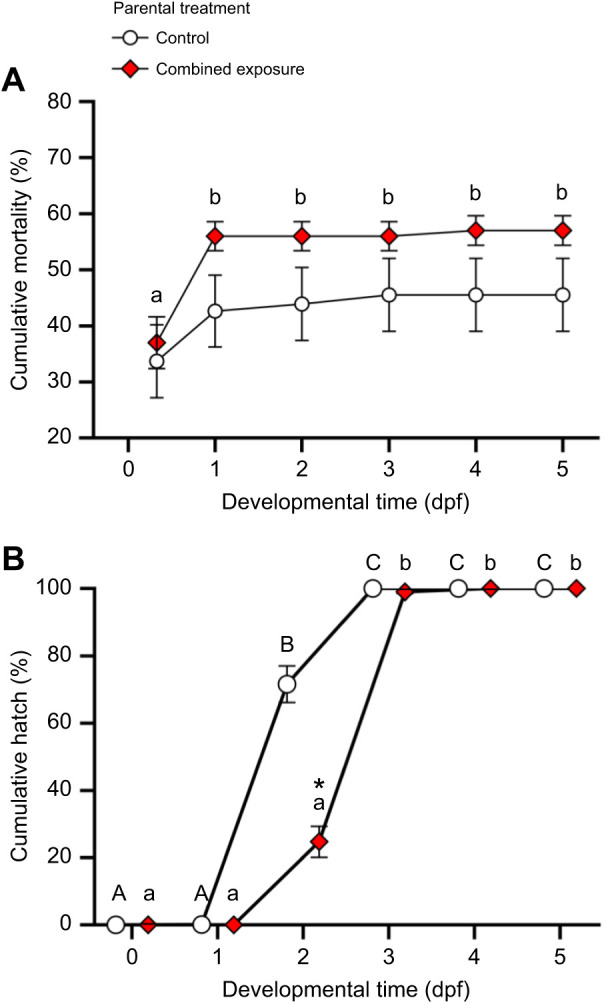
**Effects of parental treatment on zebrafish offspring cumulative mortality and cumulative hatch.** (A) Cumulative mortality; (B) cumulative hatch. Offspring were derived from adult zebrafish exposed to either control or combined exposure conditions for 14 days. Statistical differences between values were determined with Kruskal–Wallis one-way ANOVAs followed by Tukey *post hoc* tests [across developmental time (dpf); A: control: *P*=0.485; combined exposure: *P*=0.005; B: control: *P*<0.001; combined exposure: *P*<0.001], and between parental treatments with Mann–Whitney rank sum tests (A: 8 hpf: *P*=0.250; 1 dpf: *P*=0.034; 2 dpf: *P*=0.041; 3 dpf: *P*=0.060; 4 dpf: *P*=0.060; 5 dpf: *P*=0.060; B: 0 dpf: *P*=1.000; 1 dpf: *P*=0.342; 3 dpf: *P*=0.040; 4 dpf: *P*=1.000; 5 dpf: *P*=1.000) or two-tailed *t*-tests (B: 2 dpf: *P*<0.001). Assessment of each treatment group was performed at the same developmental time but are presented offset for presentation clarity. Values within a given treatment that do not share a common letter are different from one another. Differences between treatments for a given developmental time are indicated by an asterisk. Data are means±s.e.m. (*n*=20 for each time point for both treatments, for both graphs).

Although cumulative mortality did not change between 8 hpf and 5 dpf in the control treatment, it increased 1.5-fold between 8 hpf and 1 dpf in the combined exposure treatment and remained stable thereafter (Kruskal–Wallis one-way ANOVAs, control: *H*_5_=4.47, *P*=0.485; combined exposure: *H*_5_=16.57, *P*=0.005; Fig. 1A). However, with α=0.008 to account for multiple comparisons, offspring mortality did not differ between parental treatments at any given developmental time (Mann–Whitney rank sum tests, 8 hpf: *U*=157.00, *n*_1_=*n*_2_=20, *P*=0.250; 1 dpf: *U*=121.00, *n*_1_=*n*_2_=20, *P*=0.034; 2 dpf: *U*=124.00, *n*_1_=*n*_2_=20, *P*=0.041; 3 dpf: *U*=130.00, *n*_1_=*n*_2_=20, *P*=0.060; 4 dpf: *U*=130.00, *n*_1_=*n*_2_=20, *P*=0.060; 5 dpf: *U*=130.00, *n*_1_=*n*_2_=20, *P*=0.060). Finally, although cumulative hatch increased with developmental time in both treatments (Kruskal–Wallis one-way ANOVAs, control: *H*_5_=115.26, *P*<0.001; combined exposure: *H*_5_=113.06, *P*<0.001; Fig. 1B), it was ∼65% lower at 2 dpf in the offspring derived from combined exposure parents (two-tailed *t*-test, α=0.008 to account for multiple comparisons, 2 dpf: *t*_38_=6.59, *P*<0.001).

#### Offspring endocrine stress response

**Fig. 2.**
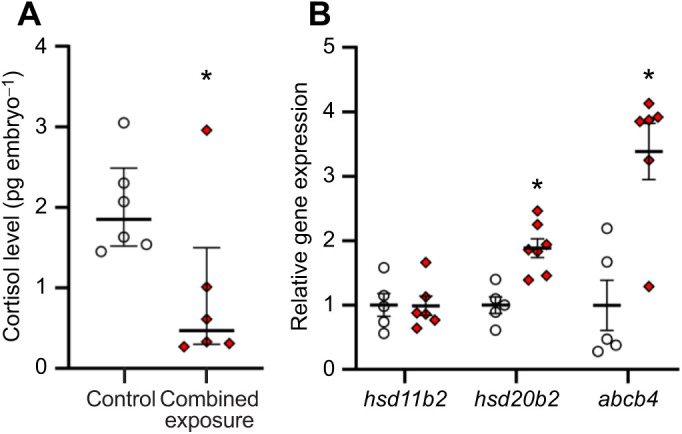
**Effects of parental treatment on zebrafish embryo endocrine stress response.** (A) Cortisol levels and (B) *hsd11b2*, *hsd20b2* and *abcb4* relative gene expression of ∼1 h post-fertilization (hpf) embryos derived from adult zebrafish exposed to either control or combined exposure conditions for 14 days. Cortisol levels were compared with a Mann–Whitney rank sum test (*P*=0.041). The gene expression values were normalized to the geometric mean of *ef1α* and *rpl13a* and the expression ratio for each gene is presented relative to the control treatment. Statistical differences between values were determined by two-tailed *t*-tests (*hsd11b2*: *P*=0.965; *hsd20b2*: *P*=0.002; *abcb4*: *P*=0.003). Differences between treatments for a given parameter are indicated by an asterisk. Data are medians and interquartile ranges in A and means±s.e.m. in B (*hsd11b2*, *hsd20b2* and *abcb4*, *n*=5–7; cortisol, *n*=6).

**Fig. 3.**
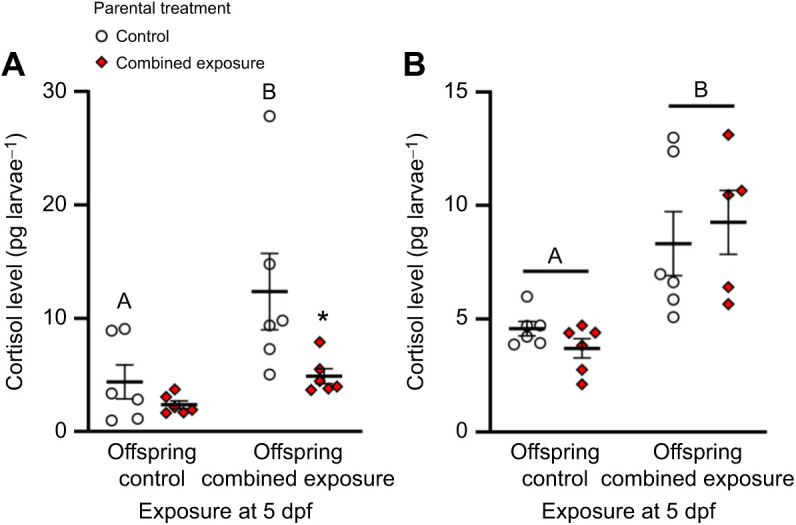
**Effects of parental treatment on zebrafish larvae cortisol stress response.** Cortisol levels after a 20 min exposure to (A) combined elevated temperature and hypoxia (offspring combined exposure), (B) a swirling stressor (offspring swirl) or control conditions (offspring control) in 5 days post-fertilization (dpf) larvae derived from adult zebrafish exposed to either control or combined exposure conditions for 14 days. Cortisol levels were compared with two-way ANOVAs followed by *post hoc* Holm–Šidák tests (A: square-root transformed, parental exposure: *P*=0.014; larval exposure: *P*=0.002; parental exposure×larval exposure: *P*=0.205; B: parental exposure: *P*=0.973; larval exposure: *P*<0.001; parental exposure×larval exposure: *P*=0.374). Differences within treatments across exposure at 5 dpf are indicated by different letters. Differences between treatments for a given exposure at 5 dpf are indicated by an asterisk. Data are means±s.e.m. (all *n*=6; except combined exposure larvae, swirl, where *n*=5).

Embryos from combined exposure parents had cortisol levels that were 54.7% lower than those from control parents (Mann–Whitney rank sum test, *U*=5.00, *n*_1_=*n*_2_=6, *P*=0.041; Fig. 2A). Although *hsd11b2* mRNA levels did not differ between treatment groups (two-tailed *t*-test, *t*_9_=0.0455, *P*=0.965; Fig. 2B), embryonic *hsd20b2* and *abcb4* mRNA levels in the combined exposure treatment were 1.9- and 3.4-fold higher, respectively, than those in the control treatment (two-tailed *t*-tests, *hsd20b2*, *t*_10_=4.32, *P*=0.002; *abcb4*, *t*_9_=–3.99, *P*=0.003). At 5 dpf, while resting cortisol levels did not differ between parental treatments, offspring exposure to a combined high temperature and hypoxia stressor increased cortisol levels by 2.8-fold in larvae derived from control parents, and had no effect on larvae derived from combined exposure parents (two-way ANOVA, square-root transformed, parental exposure: *F*_1,20_=7.17, *P*=0.014; larval exposure: *F*_1,20_=12.68, *P*=0.002; parental exposure×larval exposure: *F*_1,20_=1.71, *P*=0.205; Fig. 3A). In response to a novel swirling stressor, larvae from control and combined exposure parents increased cortisol levels by 1.8- and 2.5-fold, respectively, and there was no difference between parental treatments (two-way ANOVA, parental exposure: *F*_1,19_=0.001, *P*=0.973; larval exposure: *F*_1,19_=21.86, *P*<0.001; parental exposure×larval exposure: *F*_1,19_=0.83, *P*=0.374; Fig. 3B).

**Fig. 4.**
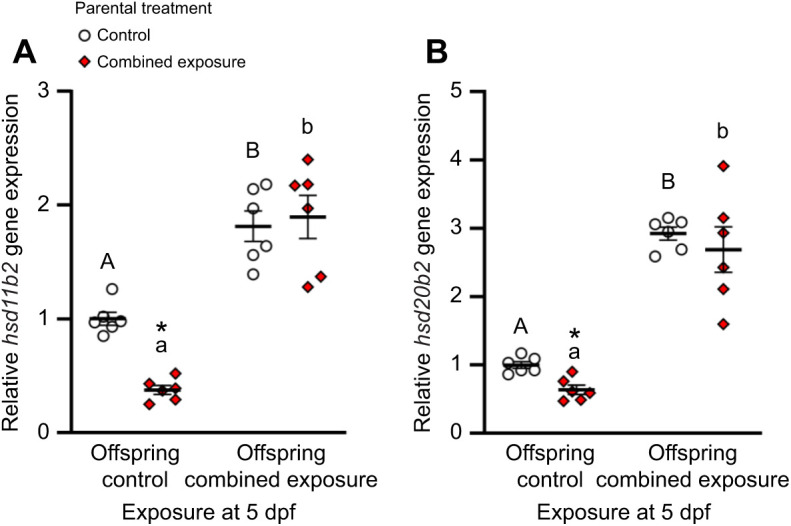
**Effects of parental treatment on zebrafish larvae endocrine stress gene expression.** (A) *hsd11b2* and (B) *hsd20b2* relative gene expression of control (offspring control) or post-exposure to combined elevated temperature and hypoxia (offspring combined exposure) 5 days post-fertilization (dpf) larvae derived from adult zebrafish exposed to either control or combined exposure conditions for 14 days. Gene expression data were normalized and expressed as stated in Fig. 2. All gene expression was further normalized to control larvae derived from control parents. Statistical differences between values were determined with two-way ANOVAs followed by *post hoc* Holm–Šidák tests (A: square-root transformed, parental exposure: *P*=0.001; larval exposure: *P*<0.001; parental exposure×larval exposure: *P*<0.001; B: log_10_-transformed; parental exposure: *P*=0.003; larval exposure: *P*<0.001; parental exposure×larval exposure: *P*=0.059). Differences within treatments across exposure at 5 dpf are indicated by different letters. Differences between treatments for a given exposure at 5 dpf are indicated by an asterisk. Data are means±s.e.m. (all *n*=6).

Although larvae derived from combined exposure parents had resting *hsd11b2* mRNA levels that were 62% lower than those from control parents, offspring exposure to a combined high temperature and hypoxia stressor increased *hsd11b2* expression to similar levels (two-way ANOVA, square-root transformed, parental exposure: *F*_1,20_=14.21, *P*=0.001; larval exposure: *F*_1,20_=129.68, *P*<0.001; parental exposure×larval exposure: *F*_1,20_=18.38, *P*<0.001; Fig. 4A). Similarly, although larvae derived from combined exposure parents had resting *hsd20b2* mRNA levels that were 36% lower than those from control parents, offspring exposure to a combined high temperature and hypoxia stressor increased *hsd20b2* expression to similar levels (two-way ANOVA, log_10_-transformed, parental exposure: *F*_1,20_=11.45, *P*=0.003; larval exposure: *F*_1,20_=205.80, *P*<0.001; parental exposure×larval exposure: *F*_1,20_=4.00, *P*=0.059; Fig. 4B).

#### Offspring cellular stress response

**Fig. 5.**
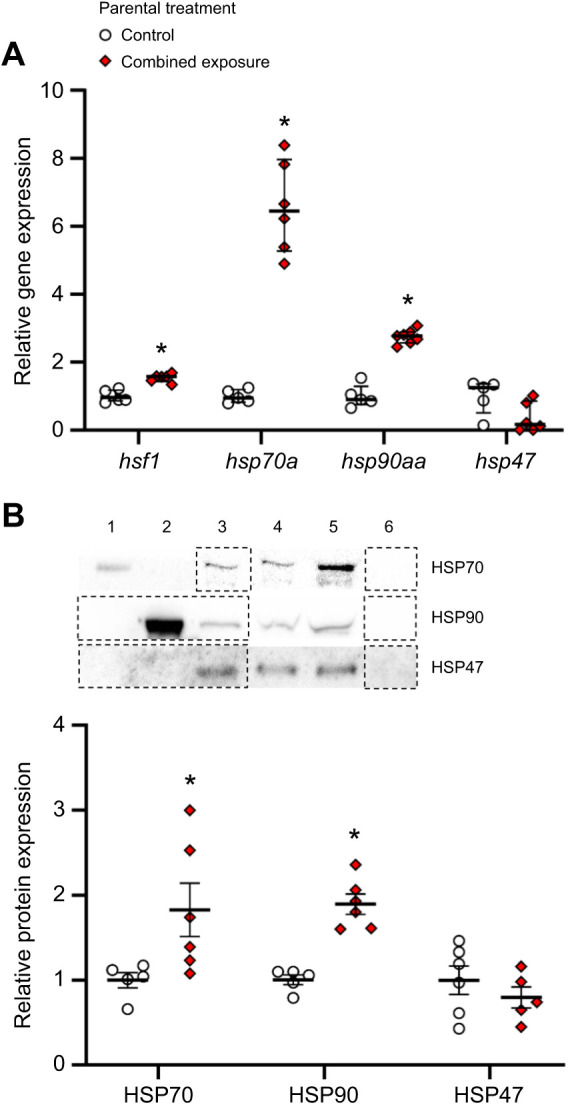
**Effects of parental treatment on zebrafish embryo cellular stress response.** (A) *hsf1*, *hsp70a*, *hsp90aa* and *hsp47 r*elative gene expression and (B) representative western blot and HSP70, HSP90 and HSP47 relative protein expression in ∼1 h post-fertilization (hpf) embryos derived from adult zebrafish exposed to either control or combined exposure conditions for 14 days. Gene expression data were normalized and expressed as stated in Fig. 2. Statistical differences between gene values were determined by two-tailed *t*-tests (*hsf1*: *P*<0.001, *hsp90aa*: *P*<0.001, *hsp47*: *P*=0.054) or a Mann–Whitney rank sum test (*hsp70a*: *P*=0.004). Representative western blot analysis of HSP70 standard (lane 1), HSP90 standard (lane 2), pool of heat stressed gills (positive control; lane 3), control treatment (lane 4), combined exposure treatment (lane 5) and blank (lane 6). Each western blot run contained multiple samples from each treatment group, but for the sake of presentation clarity, only a single replicate for each treatment is presented, requiring spliced images (represented by dashed lines). Protein expression was normalized to Coomassie stain band intensity and expressed relative to the control treatment for each protein. All statistical differences between protein values were determined by two-tailed *t*-tests (HSP70: *P*=0.046; HSP90: *P*<0.001; HSP47: *P*=0.374). Differences between treatments for a given parameter are indicated by an asterisk. Data are medians and interquartile ranges in A and are means±s.e.m. in B (*hsf1*, *hsp70a*, *hsp90aa* and *hsp47*, *n*=5–7; HSP70, HSP90 and HSP47, *n*=5–6).

Relative to embryos from control parents, those derived from combined exposure parents had 1.5-fold higher *hsf1* (two-tailed *t*-test, *t*_10_=6.40, *P*<0.001; Fig. 5A), 6.6-fold higher *hsp70a* (Mann–Whitney rank sum test, *U*=0.00, *n*_1_=5, *n*_2_=6, *P*=0.004), 2.8-fold higher *hsp90aa* (two-tailed *t*-test, *t*_10_=–11.25, *P*<0.001) and 64% lower *hsp47* mRNA levels (two-tailed *t*-test, *t*_9_=2.210, *P*=0.054). Embryonic HSP protein expression largely mirrored embryonic HSP transcript abundance. Relative to embryos from control parents, those derived from combined exposure parents had 1.8-fold higher HSP70 (two-tailed *t*-test, *t*_9_=–2.32, *P*=0.046; Fig. 5B), 1.9-fold higher HSP90 (*t*_9_=–6.34, *P*<0.001) and similar HSP47 (*t*_9_=0.94, *P*=0.374) protein abundance.

**Fig. 6.**
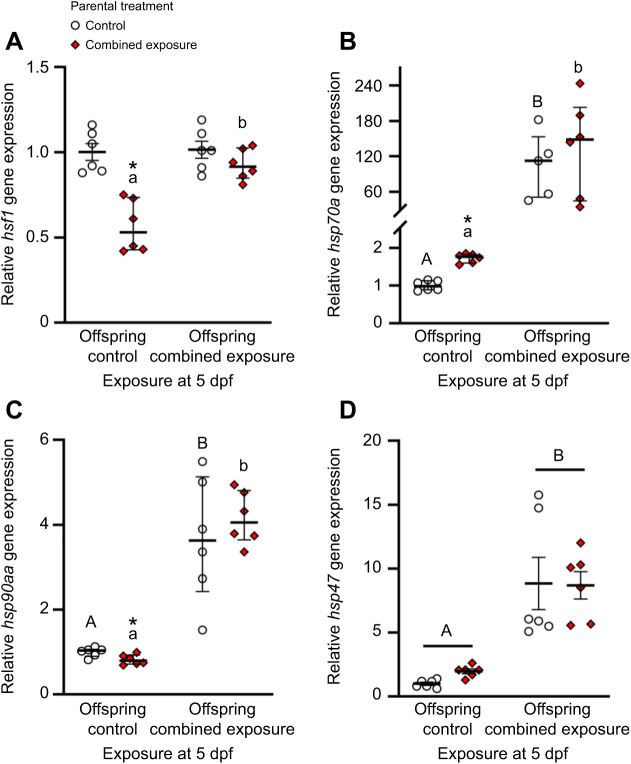
**Effects of parental treatment on zebrafish larvae cellular stress gene expression.** Control and post-exposure to combined elevated temperature and hypoxia relative gene expression for (A) *hsf1*, (B) *hsp70a*, (C) *hsp90aa* and (D) *hsp47* in 5 days post-fertilization (dpf) larvae derived from adult zebrafish exposed to exposed to either control (offspring control) or combined exposure conditions (offspring combined exposure) for 14 days. Gene expression data were normalized and expressed as stated in Fig. 4. Statistical differences between values were determined by a two-way ANOVA followed by a Holm–Šidák *post hoc* test (D: square-root transformed, parental exposure: *P*=0.266; larval exposure: *P*<0.001; parental exposure×larval exposure: *P*=0.348), or two-tailed *t*-test (A: within control: *P*=0.830, within combined exposure: *P*<0.001) or Mann–Whitney rank sum tests (B: within control: *P*=0.004, within combined exposure: *P*=0.002; C: within control: *P*=0.002, within combined exposure: *P*=0.002) across exposure at 5 dpf, followed by two-tailed *t*-test (A: offspring combined exposure: *P*=0.174; B: offspring control: *P*<0.001, offspring combined exposure: *P*=0.484; C: offspring control: *P*=0.019, offspring combined exposure: *P*=0.477) or Mann–Whitney rank sum tests (A: offspring control: *P*=0.002) between parental treatments. Differences within treatments across exposure at 5 dpf are indicated by different letters. Differences between treatments for a given exposure at 5 dpf are indicated by an asterisk. Data are medians and interquartile ranges in A–C, and means±s.e.m. in D (all *n*=6; except *hsp70a* for combined exposure larvae from control parents, *n*=5).

At 5 dpf, resting *hsf1* expression was 44% lower in larvae from combined exposure parents, and following offspring exposure to the combined high temperature and hypoxia stressor, only larvae derived from combined exposure parents increased *hsf1* mRNA levels 1.7-fold (two-tailed *t*-tests, within control: *t*_10_=–0.22, *P*=0.830, within combined exposure: *t*_10_=–4.95, *P*<0.001, offspring combined exposure: *t*_10_=1.47, *P*=0.174; Mann–Whitney rank sum test, offspring control: *U*=0.00, *n*_1_=*n*_2_=6, *P*=0.002; Fig. 6A). Unlike *hsf1*, resting *hsp70a* expression was 1.7-fold higher in 5 dpf larvae from combined exposure parents compared with larvae from control parents, and offspring exposure to the combined high temperature and hypoxia stressor increased *hsp70a* mRNA levels 105- and 78-fold in larvae derived from the control and combined exposure parental treatments, respectively (two-tailed *t*-tests, offspring control: *t*_10_=–9.98, *P*<0.001, offspring combined exposure: *t*_9_=–0.73, *P*=0.484; Mann–Whitney rank sum test, within control: *U*=0.00, *n*_1_=5, *n*_2_=6, *P*=0.004, within combined exposure: *U*=0.00, *n*_1_=*n*_2_=6, *P*=0.002; Fig. 6B). Resting *hsp90aa* expression was 18% lower in larvae from combined exposure parents than control parents, but following offspring exposure to the combined high temperature and hypoxia stressor, larvae derived from the control and combined exposure parental treatments increased *hsp90aa* mRNA levels 3.7- and 5.1-fold, respectively (two-tailed *t*-tests, offspring control: *t*_10_=2.79, *P*=0.019, offspring combined exposure: *t*_10_=–0.74, *P*=0.477; Mann–Whitney rank sum test, within control: *U*=0.00, *n*_1_=*n*_2_=6, *P*=0.002, within combined exposure: *U*=0.00, *n*_1_=*n*_2_=6, *P*=0.002; Fig. 6C). Lastly, there were no differences in resting *hsp47* expression between parental treatments, and larvae derived from the control and combined exposure parental treatments increased *hsp47* transcripts 8.8- and 4.4-fold in response to the offspring combined high temperature and hypoxia stressor, respectively (two-way ANOVA, square-root transformed, parental exposure: *F*_1,20_=1.31, *P*=0.266; larval exposure: *F*_1,20_=78.43, *P*<0.001; parental exposure×larval exposure: *F*_1,20_=0.92, *P*=0.348; Fig. 6D).

**Fig. 7.**
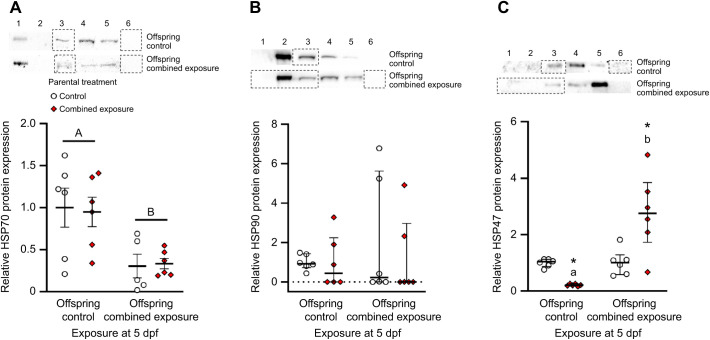
**Effects of parental treatment on zebrafish larvae cellular stress protein expression.** Control and post-exposure to combined elevated temperature and hypoxia representative western blots and relative protein expression for (A) HSP70, (B) HSP90 and (C) HSP47 in 5 days post-fertilization (dpf) larvae derived from adult zebrafish exposed to either control (offspring control) or combined exposure conditions (offspring combined exposure) for 14 days. Western blot bands and protein expression was normalized and expressed as stated in Fig. 5. All protein expression was further normalized to control larvae derived from control parents. Statistical differences between values were determined by two-way ANOVA followed by a Holm–Šidák *post hoc* test (A: parental exposure: *P*=0.948; larval exposure: *P*<0.001; parental exposure×larval exposure: *P*=0.815), or two-tailed *t*-test (C: within control: *P*=0.941) or Mann–Whitney rank sum test (B: within control: *P*=0.394, within combined exposure: *P*=0.937; C: within combined exposure: *P*=0.002) across exposure at 5 dpf, followed by two-tailed *t*-tests (B: offspring control: *P*=0.987, C: offspring Control: *P*<0.001, offspring combined exposure: *P*=0.015) or Mann–Whitney rank sum tests (B: offspring combined exposure: *P*=0.394) between parental treatments. Differences within treatments across exposure at 5 dpf are indicated by different letters. Differences between treatments for a given exposure at 5 dpf are indicated by an asterisk. Data are means±s.e.m. in A, and medians and interquartile ranges in B and C (all *n*=6, except HSP70 for combined exposure larvae from control parents*, n*=5).

At the protein level, HSP70 expression did not differ between the 5 dpf larvae derived from either parental treatment, and decreased by 69% and 64% in the larvae derived from control and combined exposure parental treatments, respectively, following offspring exposure to the combined high temperature and hypoxia stressor (two-way ANOVA, parental exposure: *F*_1,19_=0.004, *P*=0.948; larval exposure: *F*_1,19_=15.34, *P*<0.001; parental exposure×larval exposure: *F*_1,19_=0.057, *P*=0.815; Fig. 7A). HSP90 expression did not differ between either parental treatments or offspring exposures (two-tailed *t*-test, offspring control: *t*_10_=–0.02, *P*=0.987; Mann–Whitney rank sum tests, within control: *U*=12.00, *n*_1_=*n*_2_=6, *P*=0.394, within combined exposure: *U*=17.00, *n*_1_=*n*_2_=6, *P*=0.937, offspring combined exposure: *U*=12.00, *n*_1_=*n*_2_=6, *P*=0.394; Fig. 7B). Lastly, resting HSP47 expression was 78% lower in larvae derived from combined exposure than control parents, and offspring exposure to the combined high temperature and hypoxia stressor increased HSP47 by 12.9-fold in the combined exposure parental treatment, but had no effect on the larvae from control parents (two-tailed *t*-tests, within control: *t*_10_=–0.08, *P*=0.941, offspring control: *t*_10_=12.78, *P*<0.001, offspring combined exposure: *t*_[10]_=-2.92, *P*=0.015; Mann–Whitney rank sum test, within combined exposure: *U*=0.00, *n*_1_=*n*_2_=6, *P*=0.002; Fig. 7C).

#### Parental and offspring heat and hypoxia tolerance

**Fig. 8.**
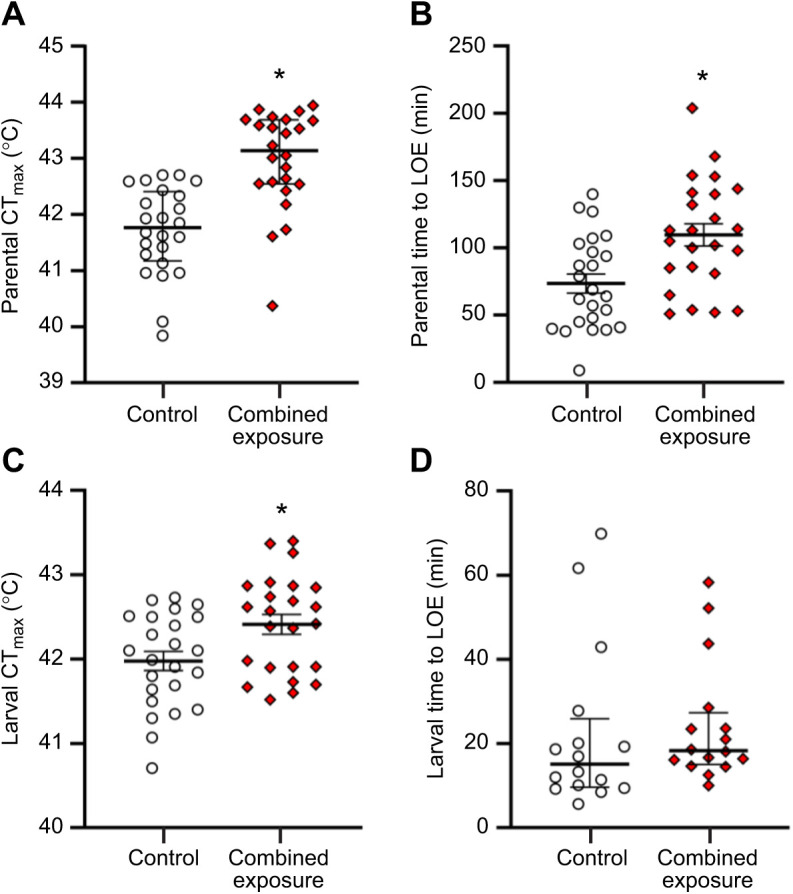
**Effects of parental treatment on parental adult and larval offspring stress tolerance.** (A) Critical thermal maxima (CT_max_) and (B) hypoxic time to loss of equilibrium (LOE) of parental adult zebrafish exposed to either control or combined exposure conditions for 14 days, and (C) CT_max_ and (D) hypoxic time to LOE for 5 days post-fertilization (dpf) larvae derived from adult zebrafish exposed to either control or combined exposure conditions for 14 days. Tolerance values were compared with Mann–Whitney rank sum tests (A: *P*<0.001; D: *P*=0.235) or two-tailed *t*-tests (B: *P*=0.002; C: *P*=0.011). Differences between treatments for a given parameter are indicated by an asterisk. Data are medians and interquartile ranges in A and D and means±s.e.m. in B and C (all *n*=24, except larvae time to LOE, *n*=16).

The CT_max_ of parents in the combined exposure treatment was 1.26°C higher than that of parents in the control treatment (Mann–Whitney rank sum test, *U*=79.00, *n*_1_=*n*_2_=24, *P*<0.001; Fig. 8A). Chronic exposure to cycling elevated temperature and hypoxia also increased parental time to LOE under hypoxic conditions by 1.5-fold (two-tailed *t*-test, *t*_46_=–3.33, *P*=0.002; Fig. 8B). Overall, males and females within a given treatment did not differ in either CT_max_ (control: males: 41.6±0.2°C, females: 41.8±0.2°C, two-tailed *t*-test, *t*_22_=0.432, *P*=0.670; combined exposure: males: 42.9±0.2°C, females: 43.0±0.3°C, Mann–Whitney rank sum test, *U*=62.50, *n*_1_=*n*_2_=12, *P*=0.603) or time to LOE (control: males: 81.0±12.1 min, females: 66.1±7.1 min, two-tailed *t*-test, *t*_22_=–1.061, *P*=0.300; combined exposure: males: 105.9±14.2 min, females: 113.3±8.9 min, two-tailed *t*-test, *t*_22_=0.438, *P*=0.666).

Larvae derived from combined exposure parents also had increased heat tolerance, as shown by a 0.44°C higher reduced movement temperature (Mann–Whitney rank sum test, *U*=138.00, *n*_1_=*n*_2_=24, *P*=0.002; Fig. S3) and a 0.43°C higher CT_max_ (two-tailed *t*-test, *t*_46_=–2.64, *P*=0.011; Fig. 8C). However, there was no difference between parental treatments for larval time to LOE when exposed to hypoxia (Mann–Whitney rank sum test, *U*=96.00, *n*_1_=*n*_2_=16, *P*=0.235; Fig. 8D). Using a 1.5×inter-quartile range test, three outliers were detected in each treatment for larval time to LOE when exposed to hypoxia. However, there was still no difference between parental treatments for larval time to LOE after removal of the outliers (two-tailed *t*-test, *t*_24_=–1.81, *P*=0.083).

## DISCUSSION

Building upon our observation that chronic exposure to environmentally relevant diel cycles of thermal stress and hypoxia affects parental progeny investment of cortisol and HSPs (Lim and Bernier, 2022), the present study provides novel evidence in fish that parental exposure to this combined stressor can affect progeny stress responsiveness and tolerance. Consistent with the environmental/maternal-matching hypothesis (Sheriff and Love, 2013), we observed that zebrafish larvae derived from parents exposed to diel cycles of heat stress and hypoxia had a reduced endocrine stress response to the combined effects of an acute heat and hypoxia challenge, as well as a higher thermal tolerance. In contrast, the same progeny had an equal or greater cellular stress response to the combined acute challenge and their hypoxia tolerance did not differ from control larvae. Overall, our study demonstrates that intergenerational plasticity in response to the combined effects of heat waves and hypoxic conditions in aquatic environments has potential benefits and limitations.

### Effects of parental exposure to cycling elevated temperatures and hypoxia

#### Parental fecundity, offspring size, cumulative mortality and cumulative hatch

Parental exposure to combined cycles of thermal stress and hypoxia had no effect on fecundity, but transiently reduced offspring size. The lack of effect of cycling thermal stress and hypoxia on zebrafish fecundity agrees with our previous observation (Lim and Bernier, 2022) and suggests that in response to a combined thermal and hypoxic challenge, the propensity of elevated temperatures below species-specific thresholds to increase clutch size in fishes are countered by the inhibitory effects of hypoxia (Wu, 2009; Ho and Burggren, 2012; Alix et al., 2020). Consistent with the inhibitory effects of parental exposure to elevated temperatures or hypoxia on egg size (Ho and Burggren, 2012; Shama and Wegner, 2014; Alix et al., 2020), we provide novel evidence that combined parental thermal stress and hypoxia can reduce egg size in fish. At 1 hpf, as previously observed in response to parental hypoxic exposure (Ho and Burggren, 2012), the reduction in egg size was not accompanied by a change in yolk size. In contrast, treated parents produced smaller 8 hpf embryos, which also had a smaller yolk size. In general, larger eggs and yolk reserves produce larger larval fish with higher survival (Chambers and Leggett, 1996; Einum and Fleming, 2000; Heath et al., 2003). Hence, the negative effects of parental thermal stress and hypoxia exposure on egg size may reduce offspring fitness. Yet, the smaller 8 hpf combined treatment embryos did not grow to become smaller larvae, a result perhaps explained by the smallest dual stressor embryos experiencing higher mortality.

Offspring derived from parents exposed to thermal stress and hypoxia were also characterized by increased mortality and delayed hatch. The marked increased in cumulative mortality between 8 hpf and 1 dpf in the combined treatment suggest that parental exposure to combined heat stress and hypoxia affects progeny gastrulation, a key developmental stage for cellular movement and differentiation which occurs between 5.5 and 10 hpf in zebrafish (Kimmel et al., 1995). Gastrulation is a critical window of thermal sensitivity in fishes, and high temperatures during this developmental stage increases the frequency of mutations, embryonic damage, and mortality (Mueller et al., 2015; Uchida et al., 2018; Bloomer et al., 2022). Moreover, gastrulation defects in zebrafish can inhibit the development of the hatching gland (Solnica-Krezel et al., 1996) and delay hatching (Ferdous et al., 2017). Overall, gastrulation is regulated by a complex set of molecules, several of which are maternally derived (Kelly et al., 2000; Nojima et al., 2004; Pei et al., 2007). Therefore, future research is needed to determine whether parental exposure to combined thermal stress and hypoxia affects the maternal deposition of gastrulation determinants. Since blocking cortisol synthesis or glucocorticoid receptor antagonism can also delay hatching in zebrafish (Wilson et al., 2013), an alternate mechanism for the observed hatch delay in the combined stressor embryos may be the reduced maternal cortisol deposition that characterized this treatment. Given that transgenerational plasticity can be maladaptive if the offspring are exposed to environments that are mismatched relative to that of their parents (Munday, 2014; Schade et al., 2014; Bautista and Burggren, 2019), future studies are also needed to establish whether the increased mortality, reduced size, and delayed hatch of the combined stressor embryos are context dependent.

#### Offspring endocrine stress response

Our study suggests the existence of barriers against parental progeny cortisol deposition in response to diel cycles of elevated temperatures and hypoxia and that this environmental challenge has programming effects on offspring cortisol catabolism. Consistent with our previous findings (Lim and Bernier, 2022), we observed that parental heat stress and hypoxia exposure reduced maternal deposition of cortisol and, as evidenced by the elevated *hsd20b2* and *abcd4* mRNA levels at 1 hpf, increased the capacity of combined exposure embryos to metabolize and excrete cortisol (Paitz et al., 2016; Faught and Vijayan, 2018). Interestingly, parental exposure to diel cycles of thermal stress and hypoxia in Lim and Bernier (2022) increased the maternal transfer of *hsd11b2* but not *hsd20b2* transcripts. These divergent results are likely due to differences in experimental conditions. The 14-day combined parental exposure of Lim and Bernier (2022) used more severe diel cycles of hypoxia (20–85% DO) than the current study (30–85% DO). Overall, with few exceptions (e.g. Stratholt et al., 1997), chronic maternal stress or elevated maternal plasma cortisol levels do not increase egg cortisol content in fishes (Mileva et al., 2011; Sopinka et al., 2014; Jeffrey and Gilmour, 2016; Taylor et al., 2016; Lim and Bernier, 2022; Magierecka et al., 2022) implying that the ovary and embryo have a high capacity to buffer against maternal cortisol transfer in these animals. Nevertheless, although basal cortisol levels did not differ between larval treatments, the reduced *hsd11b2* and *hsd20b2* resting mRNA levels of the 5 dpf combined exposure larvae suggest potential carryover effects of the parental treatment on progeny cortisol catabolism. Although confirmation of these results at the protein and functional levels are needed, this downregulation of *hsd11b2* and *hsd20b2* may be a compensatory mechanism serving to restore resting cortisol levels in larval fish to basal levels. Similarly, in the absence of maternal cortisol contribution, previous studies in zebrafish (Jeffrey and Gilmour, 2016) and sockeye salmon (Sopinka et al., 2017b) have shown that chronic maternal stress can have programming effects on the resting mRNA levels of genes involved in cortisol signaling, biosynthesis and catabolism.

As predicted, we observed that offspring derived from parents exposed to diel cycles of heat stress and hypoxia have a reduced endocrine stress response when faced with the same challenge as larval fish. Consistent with the observation that either acute heat stress (36°C for 10 min; Yeh et al., 2013) or hypoxia exposure (10% DO for 2 h; Mikloska et al., 2022) stimulates the HPI axis in 5 dpf larval zebrafish, we demonstrate that the two stressors combined (36°C and 20% DO for 20 min) elicit an increase in whole body cortisol in control larvae, but has no effect in the larvae derived from parents exposed to cycles of thermal stress and hypoxia. In contrast, the 5 dpf larvae from both parental treatments had a similar increase in whole body cortisol following a swirling stressor, and the magnitude of the response was consistent with a previous observation (Williams et al., 2017). Together, these results suggest that sustained parental exposure to cycles of thermal stress and hypoxia increase the tolerance of larvae to a similar challenge but does not affect their capacity to mount a cortisol stress response to a novel stressor. Although previous studies using 5 dpf zebrafish larvae have shown that stressors can upregulate *hsd11b2* and *hsd20b2* expression (Tokarz et al., 2013; Mikloska et al., 2022), here we provide novel evidence that acute exposure to combined heat stress and hypoxia also stimulates the expression of both transcripts. Given that *hsd11b2* and *hsd20b2* expression is cortisol-dependent (Tokarz et al., 2013) and that larvae derived from parents exposed to cycles of thermal stress and hypoxia did not mount a cortisol stress response when challenged with a similar stressor, the finding that larvae from both parental treatments upregulated these transcripts was unexpected. The larger increase in *hsd11b2* and *hsd20b2* expression in offspring from combined exposure parents may provide a mechanism through which the larval cortisol stress response can be muted when faced with a similar challenge. Overall, as previously observed in response to altered maternal social status in zebrafish (Jeffrey and Gilmour, 2016) and to chronic maternal chasing in sockeye salmon (Sopinka et al., 2017b), our results show that sustained parental exposure to diel cycles of heat stress and hypoxia can affect offspring stress responsiveness.

#### Offspring cellular stress response

In addition to promoting the embryonic deposition of inducible HSPs, parental exposure to cycles of elevated temperatures and hypoxia altered the larval basal expression of key mediators of the cellular stress response. Consistent with recent findings in zebrafish (Lim and Bernier, 2022), we observed that parental exposure to the combined stressor of high temperature and hypoxia results in a marked parental progeny investment of *hsp70a* mRNA, smaller deposits of *hsf1* and *hsp90aa* transcripts, and increases in embryonic HSP70 and HSP90 content. Of note, however, the embryonic deposition of maternal HSPs was smaller than previously observed and did not include *hsp47* transcripts (Lim and Bernier, 2022), differences most likely explained by the less severe diel cycles of hypoxia used in this study. At the larval stage, epigenetic mechanisms may be responsible for the altered basal expression of HSPs observed in this study. Although the specific role of epigenetics in the intergenerational plasticity of molecular chaperones in response to environmental stressors is yet unclear, various HSPs are known methylation targets (Venney et al., 2016; Weyrich et al., 2016) and exposure to stressors can alter the basal expression of HSPs and confer epigenetic responses across generations (Norouzitallab et al., 2014; Luu et al., 2021). Alternatively, since molecular chaperones have dynamic temporal expression profiles during development (Krone et al., 1997, 2003), the altered basal expression of HSPs in larvae may have resulted from the delayed hatch of the combined stressor progeny. Regardless of its cause, given the pleiotropic functions of HSPs during development and their key role in phenotypic determination (Queitsch et al., 2002; Yeyati et al., 2007; Chen et al., 2018), our results lead to the possibility that HSPs underlie some of the capacity for intergenerational tolerance to environmental stressors.

Contrary to our prediction, zebrafish larvae derived from parents exposed to diel cycles of heat stress and hypoxia did not have a blunted cellular stress response to a combined elevated temperature and hypoxia challenge. The stimulatory effects of the acute thermal and hypoxia combined stressor on *hsp70a*, *hsp90aa* and *hsp47* transcript levels in the control 5 dpf larvae is consistent with the known effects of these individual stressors on HSP transcription in zebrafish (Krone and Sass, 1994; Lele et al., 1997; Ton et al., 2003; Levesque et al., 2019; Lim and Bernier, 2022) and in other fish species (Fuzzen et al., 2011; Narum et al., 2013; Wang et al., 2016; Mackey et al., 2021). Our observation that the larval induction of *hsp70a*, *hsp90aa* and *hsp47* in response the combined stressor is independent of the parental treatment, suggest that sustained parental exposure to cycles of thermal stress and hypoxia does not reduce the cellular damage caused by a similar challenge in their offspring or reprogram the larval transcriptional machinery of the heat shock response. At the protein level, despite evidence from several studies that heat shock or hypoxia exposure can increase HSP70 in adult fish (Todgham et al., 2005; Currie et al., 2010; LeBlanc et al., 2011; Chadwick and McCormick, 2017; Lim and Bernier, 2022), the acute thermal and hypoxia challenge unexpectedly decreased larval HSP70 levels in both parental treatments. Although elevated levels of cortisol can decrease the heat-stress induced levels of HSP70 and suppress basal HSP70 expression (Basu et al., 2001), a role of cortisol in repressing HSP70 translation in this study seems unlikely as the larvae derived from parents exposed to cycles of thermal stress and hypoxia did not mount a cortisol stress response to the combined stressor. In contrast, the variable larval HSP90 expression and lack of induction after the combined stressor is not surprising given that this protein is only weakly inducible with heat stress in zebrafish (Murtha and Keller, 2003; Lim and Bernier, 2022). Alternatively, as previously observed in zebrafish embryos (Connolly and Hall, 2008), the mismatch in larval HSP70 and HSP90 mRNA and protein levels may result from a developmental-stage specific differential regulation of HSP transcription and translation in response to environmental stress. Lastly, while opposite to our prediction, we note that larvae derived from parents exposed to elevated temperatures and hypoxia were characterized by a larger HSP47 induction when exposed to a similar challenge. Although HSP47 was initially identified as a collagen-specific molecular chaperone, its association with other endoplasmic reticulum-resident stress proteins suggest a broader role for HSP47 in the cellular stress response (Ito and Nagata, 2019). For example, though the mechanism is not known, thermal stress tolerance in desert populations of redband trout has been partly attributed to single nucleotide polymorphisms in the 3′ untranslated region of the *hsp47* gene (Narum et al., 2013). Similarly, whether the larger HSP47 induction of larvae derived from stress-challenged parents is an adaptive response that mitigates the cellular damage inflicted by heat stress and hypoxia, and/or increases tolerance to the combined stressor remains to be determined.

#### Parental and offspring heat and hypoxia tolerance

Our results provide novel evidence that environmentally relevant cycles of combined nocturnal hypoxia and daytime elevated temperatures can increase both thermal and hypoxia tolerance in adult zebrafish as well as larval thermal tolerance via intergenerational plasticity. In general, although there is some evidence of cross-protection between elevated temperatures and hypoxia (Rodgers and Gomez Isaza, 2021), i.e. chronic acclimation to an elevated temperature can improve hypoxia tolerance (McBryan et al., 2016; Collins et al., 2021) and vice-versa (Burleson and Silva, 2011), acclimation to combined high temperatures and hypoxia is clearly challenging for fish and has mixed effects on thermal and hypoxia tolerance (Anttila et al., 2015; Del Rio et al., 2019; McDonnell et al., 2019; Earhart et al., 2022). In this study, the beneficial effects of acclimation to combined high temperatures and hypoxia on both parental heat and hypoxia tolerance may be a physiological consequence of the fluctuating properties of the acclimation regime. Although cycling and constant elevated temperature acclimation are both well known to increase thermal tolerance in fishes (Beitinger et al., 2000; Corey et al., 2017; Cooper et al., 2021), the gene expression profile associated with fluctuating temperatures is distinct from the profile that results from acclimation to chronic high temperatures (Podrasky and Somero, 2004). Similarly, although acclimation to constant and intermittent hypoxia both increase hypoxia tolerance in killifish (*Fundulus heteroclitus*), the mechanisms involved are distinct and intermittent hypoxia appears to specifically improve the capacity to recover from hypoxic bouts (Borowiec et al., 2015). Furthermore, killifish acclimated to fluctuating temperatures demonstrate cross-tolerance, as acclimated fish had increased hypoxia tolerance across a broader range of temperatures relative to killifish acclimated to constant temperatures, and was associated with increased haemoglobin-O_2_ affinity (Ridgway and Scott, 2023).

In larval fish, consistent with the increase in thermal tolerance via intergenerational plasticity observed here, previous studies have shown that parents reared at high temperatures can not only produce offspring with increased heat tolerance and survival (Butzge et al., 2021), but also improved aerobic capacity (Donelson et al., 2012; Bernal et al., 2022) and growth performance (Salinas and Munch, 2012; Butzge et al., 2021; Chang et al., 2021; Munch et al., 2021). In marine sticklebacks (*Gasterosteus aculeatus*), offspring were larger when reared under the matching elevated temperature conditions of their mothers and the beneficial effects of transgenerational thermal acclimation on body size were linked to metabolic modifications of maternally inherited mitochondria (Shana et al., 2014). Whether similar metabolic adjustments drive the intergenerational plasticity in thermal tolerance identified in this study remains to be determined. In contrast, although parental acclimation to constant hypoxia (∼62% DO) for 2–4 weeks can increase offspring hypoxia tolerance in zebrafish (Ho and Burggren, 2012), parental exposure to combined cycles of heat stress and hypoxia in this study did not confer offspring hypoxia resistance. However, although we assessed the intergenerational influences of combined heat stress and hypoxia on offspring hypoxia tolerance in 5 dpf larvae, an increase in hypoxia tolerance via intergenerational plasticity was not observed until the larvae were 9–12 dpf in Ho and Burggren (2012). Moreover, because hypoxia resistance in control zebrafish larvae significantly decreases between 5 and 9 dpf (Ho and Burggren, 2012), we suggest that future experiments are warranted to assess whether the intergenerational effects of combined heat stress and hypoxia on offspring hypoxia tolerance depends on larval age. Overall, although our results provide evidence that acclimation to combined high temperature and hypoxia can have beneficial effects on parental and progeny resilience to environmental stressors, they also suggest that the outcome may depend on the nature of the thermal and hypoxia exposure, its severity, and the developmental stage used for testing.

In conclusion, this study provides novel evidence that parental exposure to the combined stressors of high temperature and hypoxia can affect progeny tolerance to environmental stressors and stress responsiveness. Specifically, we demonstrate that larvae derived from parents chronically exposed to diel cycles of elevated temperatures and hypoxia have an increased thermal tolerance, and when faced with a similar thermal and hypoxic challenge, a reduced cortisol stress response. Although these findings suggest that intergenerational acclimation to increases in water temperatures and hypoxic conditions may help fish acclimate to future climate change, the combined stressor embryos also had a reduced fitness, suggesting, as previously observed (Munday, 2014; Bautista and Burggren, 2019; Earhart et al., 2022), potential trade-offs to transgenerational plasticity. Moreover, it remains to be determined whether the potential benefits of intergenerational acclimation to elevated temperatures and hypoxia are maintained past the larval stage and have transgenerational effects. In addition to reducing embryo cortisol content and increasing maternal deposition of HSPs, our results demonstrate that intergenerational acclimation to high temperatures and hypoxia has programming effects on the endocrine and cellular stress responses of larvae. Therefore, although much work remains to identify the mechanisms driving intergenerational plasticity, such as the various epigenetic signatures transmitted through either the maternal or paternal lines that may contribute to offspring phenotypes (Metzger and Schulte, 2016; Perez and Lehner, 2019), we suggest that future studies are also needed to determine the roles of maternal cortisol and HSPs in shaping the capacity for intergenerational tolerance to environmental stressors.

## Supplementary Material

10.1242/jexbio.245583_sup1 Supplementary information
