# Correction: Intergenerational plasticity to cycling high temperature and hypoxia affects offspring stress responsiveness and tolerance in zebrafish

**DOI:** 10.1242/jeb.247192

**Published:** 2024-01-23

**Authors:** Michael Y.-T. Lim, Nicholas J. Bernier

There was an error in *J. Exp. Biol.* (2023) **226**, jeb245583 (doi:10.1242/jeb.245583).

Incorrect HSP70 sample blots were selected for Fig. 5B as a result of a splicing error. This has been corrected in the revised Fig. 5B. The authors have also taken this opportunity to replace representative blots of lanes 1–3 with those that require less splicing where possible. Lanes 1–3 on each blot contained an HSP70 standard (lane 1), an HSP90 standard (lane 2) and a pool of heat stressed gills (positive control; lane 3). Since the blots contained a mixture of samples from control and combined exposure treatments, and in some instances both embryo and larvae samples, representative HSP70 lanes 1–3 are shown more than once (e.g. in the revised Fig. 5B and Fig. 7A, as indicated in the revised figure legend). Revised and original versions of Fig. 5B and Fig. 7A,C are shown here.

**Fig. 5B (corrected panel):**
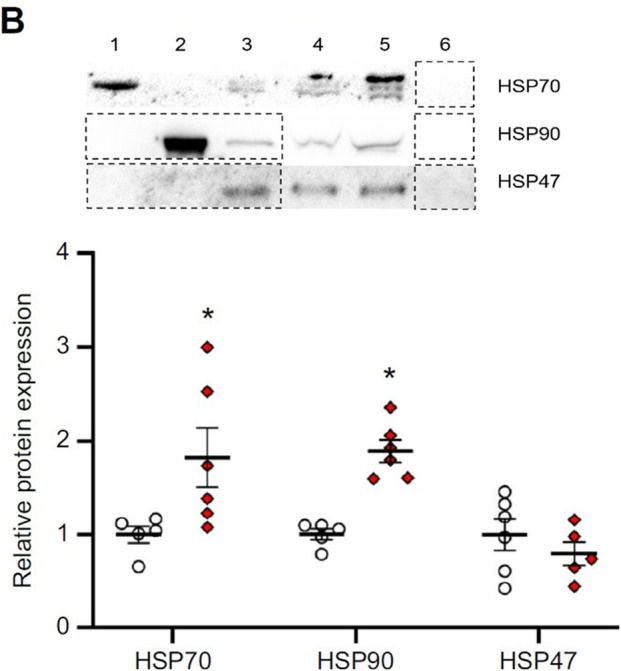
**Effects of parental treatment on zebrafish embryo cellular stress response.** (B) Representative western blot and HSP70, HSP90 and HSP47 relative protein expression in ∼1 h post-fertilization (hpf) embryos derived from adult zebrafish exposed to either control or combined exposure conditions for 14 days.

**Fig. 5B (original panel):**
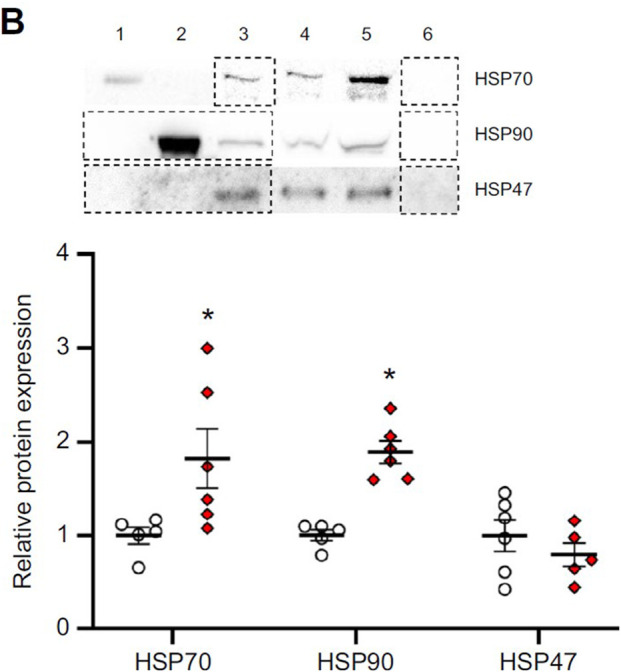
**Effects of parental treatment on zebrafish embryo cellular stress response.** (B) Representative western blot and HSP70, HSP90 and HSP47 relative protein expression in ∼1 h post-fertilization (hpf) embryos derived from adult zebrafish exposed to either control or combined exposure conditions for 14 days.

The online full text and PDF versions of the paper have been corrected. The authors apologise to readers for this error, which does not impact the statistical analysis, interpretation or conclusions of the paper.

**Fig. 7 (corrected):**
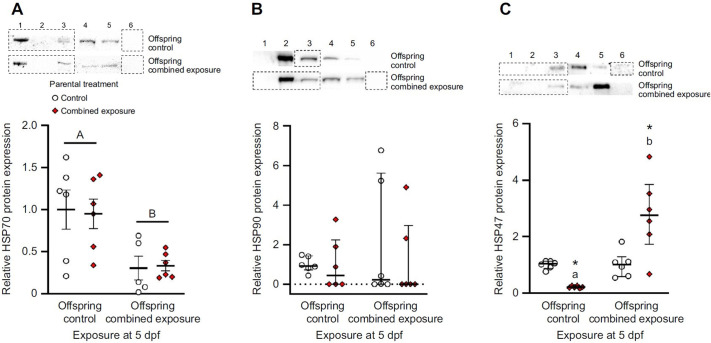
**Effects of parental treatment on zebrafish larvae cellular stress protein expression.** Control and post-exposure to combined elevated temperature and hypoxia representative Western blots and relative protein expression for (A) HSP70, (B) HSP90, and (C) HSP47 in 5 days post-fertilization (dpf) larvae derived from adult zebrafish exposed to either control (offspring control) or combined exposure conditions (offspring combined exposure) for 14 days. Western blot bands and protein expression was normalized and expressed as stated in Fig. 5. Representative HSP70 control bands (lanes 1 to 3) from Fig. 5B were re-used for Fig. 7A offspring control bands as the blot contained both embryo and larvae samples.

**Fig. 7 (original):**
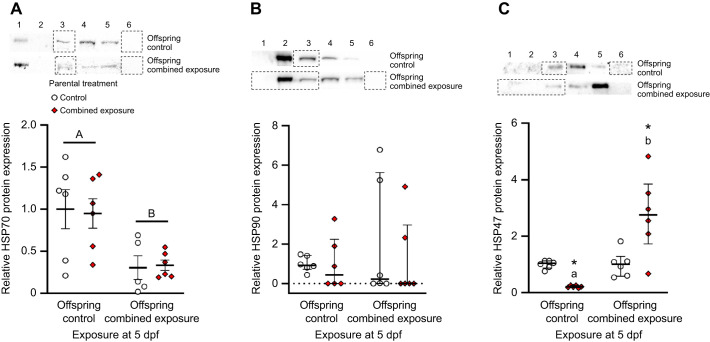
**Effects of parental treatment on zebrafish larvae cellular stress protein expression.** Control and post-exposure to combined elevated temperature and hypoxia representative Western blots and relative protein expression for (A) HSP70, (B) HSP90, and (C) HSP47 in 5 days post-fertilization (dpf) larvae derived from adult zebrafish exposed to either control (offspring control) or combined exposure conditions (offspring combined exposure) for 14 days. Western blot bands and protein expression was normalized and expressed as stated in Fig. 5.

