# Supplementary material for: Intergenerational plasticity to cycling high temperature and hypoxia affects offspring stress responsiveness and tolerance in zebrafish
Source: J Exp Biol. 2023 Aug 21;226(16):jeb245583. doi: 10.1242/jeb.245583 (PMC10482009; doi:10.1242/jeb.245583)
Supplement: Supplementary information [file jexbio-226-245583-s1.pdf]

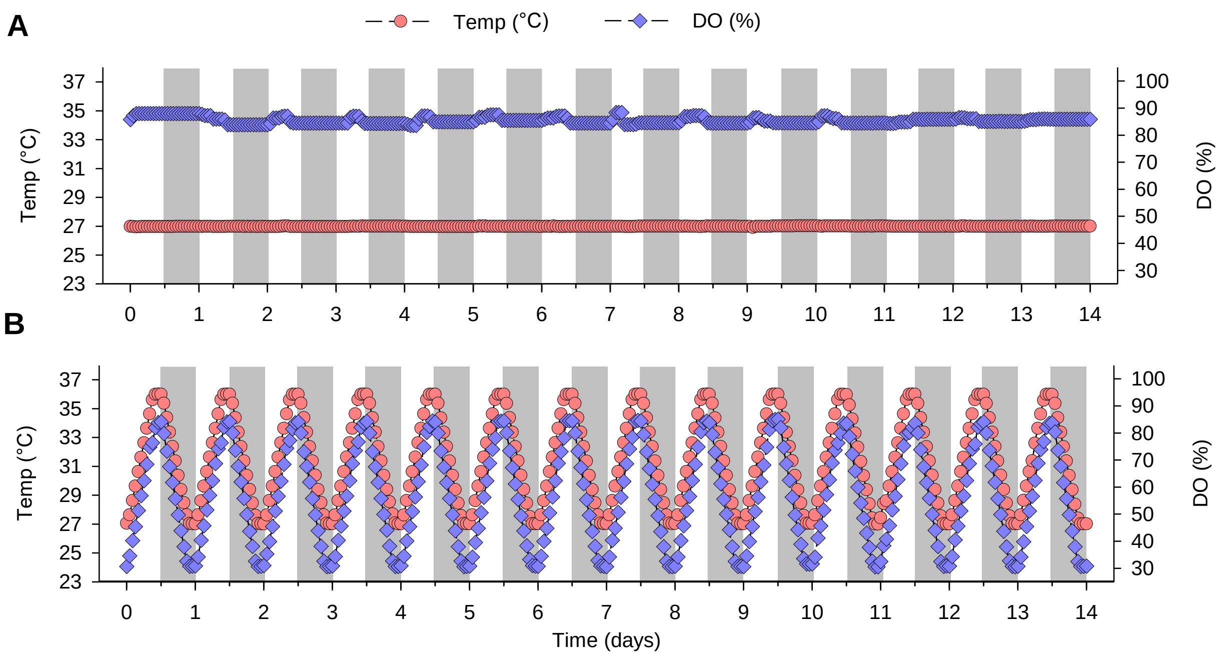

Fig. S1. Temperature (Temp) and % dissolved oxygen (DO) profiles of the (A) control and (B) combined exposure treatments. In panel (B), Temp and %DO increase during the light phase (white background) and decrease during the dark phase (gray background). Note that maximum Temp and minimum %DO levels are offset from each other by 12 h. For presentation clarity, only the 60 min running means are shown over the course of 14 days.

Journal of Experimental Biology • Supplementary information

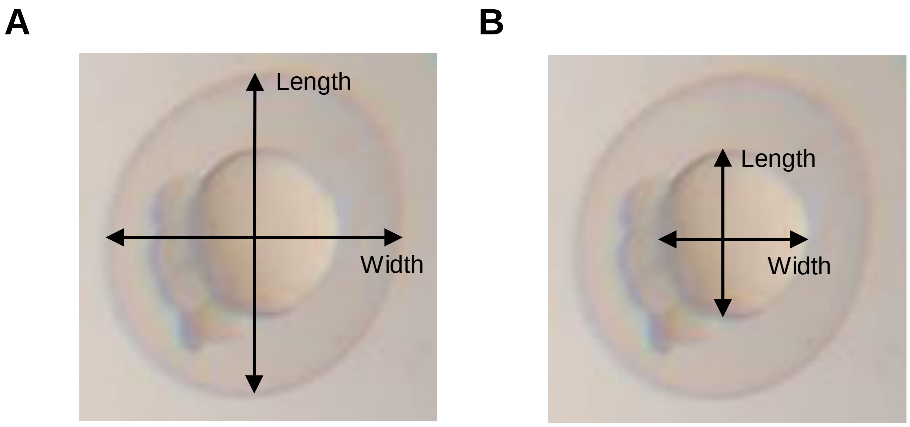

Fig. S2. Representative (A) egg size and (B) yolk size 1 hpf embryo measurements. Two perpendicular diameters (Length and Width) of the maximum cross-sectional area delimited by the (A) chorion and (B) yolk sac are shown. For presentation clarity, the same embryo is shown in (A) and (B).

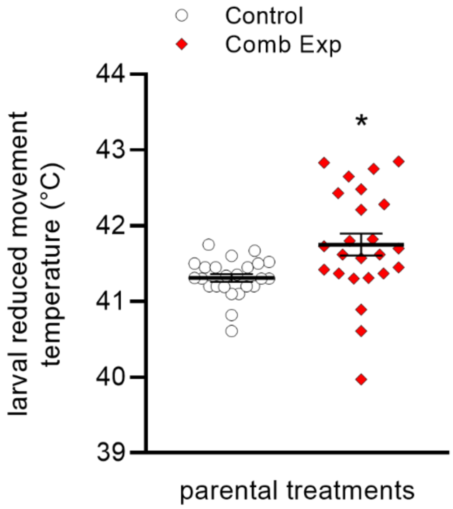

Fig. S3. Reduced movement temperature in 5 days post-fertilization (dpf) larvae derived from adult female zebrafish exposed to either control or combined exposure (Comb Exp) conditions for 14 days. Reduced movement temperature was compared with a two-tailed t-test. Differences between treatments are indicated by an asterisk. Data are mean ± s.e.m. (*n*=24 for both treatments).

Journal of Experimental Biology • Supplementary information

Table S1. Nucleotide sequences of zebrafish primers used for quantitative real-time PCR.

Gene	Accession no.	Sequence (5' – 3')	Efficiency (%)
abcb4	NM_001316714.1	F: CTGGCAGGACATGCTCTAAA<br>R: TTCTCAATGGCTTCTGTTGC	94.3
ef1α	NM_131263.1	F: GGGCAAGGGCTCCTTCAA<br>R: CGCTCGGCCTTCAGTTTG	102.2
hsd11b2	NM_212720.2	F: TGCTGCTGGCTGTACTTCAC<br>R: TGCATCCAACTTCTTTGCTG	92.7
hsd20b2	KM_279631.1	F: GCTGGGCTGTTGTAACTGGT<br>R: TTGGCAAGCTCTTCAGCATA	107.3
hsf1	NM_001313736	F: ATCACATTAGCACGGTCCAA<br>R: GAGCTGCTGTCGTTCAACAT	96.9
hsp47	NM_131204.2	F: CAATGTCTTCCATGCCTCCT<br>R: AGCTTGGGGTTCCTCATCTT	97.1
hsp70a	AF_210640	F: AAGATCACCATCACCAACGA<br>R: TGCACCATTCTCTCGATCTC	102.4
hsp90aa	NM_131328	F: CGGTTACCCAATTACGCTTT<br>R: TTTCTCGCCTTCCTCAAGAT	102
rpl13a	NM_212784.1	F: ATGCTTCCACACAAAACCAA<br>R: CATGCGCTTTCTCTTGTCAT	101.1

abc, ATP binding cassette; *bhsd*, beta hydroxysteroid dehydrogenase; *ef1α*, elongation factor 1*α*; F, forward; *hsf*, heat shock factor; *hsp*, heat shock protein; R, reverse; *rpl13a*, ribosomal protein L13A

Journal of Experimental Biology • Supplementary information
